# Transcriptome analysis reveals that auxin promotes strigolactone-induced adventitious root growth in the hypocotyl of melon seedlings

**DOI:** 10.3389/fpls.2023.1192340

**Published:** 2023-06-12

**Authors:** Jingrui Li, Mi Fan, Qinqin Zhang, Guiyun Lü, Xiaolei Wu, Binbin Gong, Yubo Wang, Ying Zhang, Hongbo Gao

**Affiliations:** ^1^ College of Horticulture, Hebei Agricultural University, Baoding, China; ^2^ Collaborative Innovation Center of Vegetable Industry in Hebei, Baoding, China; ^3^ Hebei Key Laboratory of Vegetable Germplasm Innovation and Utilization, Baoding, China

**Keywords:** melon, strigolactones, auxin, adventitious root, transcriptome, phytohormone

## Abstract

**Introduction:**

Strigolactone (SL) and auxin are two important phytohormones involved in plant root development, but whether they show synergistic or mutual promotion effects during adventitious root (AR) formation has not been adequately explored.

**Methods:**

In this study, we investigated the mechanisms of GR24 (synthetic SL) and indole-3-acetic acid (IAA; a type of auxin) in the formation of ARs using melon as the study material.

**Results:**

Morphological measurements showed that the AR number, length, superficial area, and volume under the GR24 treatment were 1.60–3.27, 1.58–3.99, 2.06–3.42, and 3.00–6.11 times greater than those of the control group, respectively, at 6–10 days; the GR24+IAA treatment further promoted AR formation in melon seedlings, and the AR number, length, superficial area, and volume under the GR24+IAA treatment were 1.44–1.51, 1.28–1.73, 1.19–1.83, and 1.31–1.87 times greater than those obtained with the GR24 treatment, respectively. Transcriptome analysis revealed 2,742, 3,352, and 2,321 differentially expressed genes (DEGs) identified from the GR24 *vs.* control, GR24+IAA *vs.* control, and GR24+IAA *vs.* GR24 comparisons, respectively. The GR24 treatment and GR24+IAA treatment affected auxin and SL synthesis as well as components of the phytohormone signal transduction pathway, such as auxin, brassinosteroid (BR), ethylene (ETH), cytokinin (CK), gibberellin (GA), and abscisic acid (ABA). The concentrations of auxin, GA, zeatin (ZT), and ABA were evaluated using high-performance liquid chromatography (HPLC). From 6 to 10 days, the auxin, GA, and ZT contents in the GR24 treatment group were increased by 11.48%–15.34%, 11.83%–19.50%, and 22.52%–66.17%, respectively, compared to the control group, and these features were increased by 22.00%–31.20%, 21.29%–25.75%, 51.76%–98.96%, respectively, in the GR24+IAA treatment group compared with the control group. Compared to that in the control, the ABA content decreased by 10.30%–11.83% in the GR24 treatment group and decreased by 18.78%-24.00% in the GR24+IAA treatment group at 6–10 days.

**Discussion:**

Our study revealed an interaction between strigolactone and auxin in the induction of AR formation in melon seedlings by affecting the expression of genes related to plant hormone pathways and contents.

## Introduction

Roots are important organs for efficiently obtaining water and nutrients for plant development and reproduction. The plant root system contains embryonic and postembryonic components (Birnbaum, 2016). Adventitious roots (ARs) are secondary roots that formed from stems, leaves, or other plant tissues, demonstrating the regenerative ability of plants. Melon (*Cucumis melo* L.) is one of the most consumed fruits in the world. In melon plants, the roots are fragile, the root zone is shallow, and the AR regeneration ability is weak. These characteristics cause plants to be easily injured as a result of seedling transplantation, double root-cutting grafting, or abiotic stresses such as waterlogging (Zhang et al., 2021), which prolong the seedling period, postpone the harvest, and even affect survival. Thus, the identification of an effective method for promoting AR growth when melon seedlings are transplanted or subject to abiotic stresses has recently become a vital goal for high-quality production.

AR development can be influenced by various external environmental factors, particularly damage, light, gravity, and internal phytohormones (Gutierrez et al., 2012; Lin and Sauter, 2019). Phytohormones such as auxin, cytokinin (CTK), ethylene, jasmonate (JA), gibberellin (GA), and abscisic acid (ABA) play important roles in the formation of ARs (Druege et al., 2016; Gonin et al., 2019).

Phytohormone levels and gene expression related to phytohormone signaling pathways show significant changes during AR formation (Guan et al., 2019; Betti et al., 2021). For example, indole-3-carboxylic acid, ABA, trans-zeatin, and JA levels change during AR formation in tea. Moreover, a transcriptome analysis of ARs under different light treatments showed that differentially expressed genes (DEGs) were significantly enriched in the plant hormone synthesis and transduction-related pathway, including *flavin monooxygenase* (*YUC*) and *auxin influx carrier* (*AUX1*) (Shen et al., 2022). In apple rootstock, the levels of the endogenous phytohormones indole-3-acetic acid (IAA), zeatin riboside, JA, GA, and ABA in the process of AR formation were changed, and DEGs were shown to be considerably enriched in hormone signaling-related pathways (Tahir et al., 2021). Cucumber AR formation in the hypocotyl is also regulated by genes related to hormone synthesis and the AR signaling pathway (Wang et al., 2022). These results prove that phytohormone synthesis and signal transduction are necessary for AR formation.

Strigolactones (SLs) are a novel class of phytohormones, derived from the catabolism of carotenoids, which are produced mainly in roots (Umehara et al., 2008). Researchers have studied the inhibitory effect of SLs on lateral shoot branching (Khuvung et al., 2022; Rani et al., 2023). SLs can also regulate seed germination, nutrient acquisition, symbiotic and parasitic interactions, and responses of plants to abiotic and biotic stresses (Ma et al., 2020). Recently, substantial progress has been made in revealing the mechanism of SLs in regulating root growth and development (Sun et al., 2022). Exogenous GR24 affects root development by affecting endogenous hormones in plants (Jiu et al., 2022). Increasing amounts of data indicate that SLs act synergistically with other plant hormones, especially auxin, to affect plant root development (Sun et al., 2022). Moreover, auxin may regulate SL biosynthesis to modulate apical dominance (Ferguson & Beveridge, 2009). However, in the process of AR formation, it is unclear whether there is a synergistic or mutually promoting relationship between SL and auxins.

It is likely that the application of exogenous phytohormones can affect endogenous phytohormone levels and then trigger the expression of genes required for AR formation. To explore the relationships between GR24 and auxin in AR formation in melon seedlings, we investigated melon hypocotyl AR growth traits, gene expression, and phytohormone levels in response to exogenous GR24 and GR24+IAA treatments. This study reveals the mechanism by which SLs and auxin exert a protective effect on AR formation in melon seedlings.

## Materials and methods

### Plant materials

Melon seeds of ‘Xizhoumi No. 25’ were placed in culture dishes with wet filter paper and then cultured in an incubator at 28°C. After the radicle sprouted, the seedlings were transplanted into a 50-hole tray with a peat:vermiculite mixture at a ratio of 2:1. Melon seedlings were cultured in a greenhouse at 27°C/18°C (day/night), with a photoperiod of 14/10 hours (day/night) and humidity of 75%.

### SL and auxin treatments

When the melon seedlings were in the four-leaf stage, the uniform seedlings were divided into three groups, and their roots were cut. The seedlings were subjected to the following hormone treatments:

1) control treatment (Control): the hypocotyls of melon seedlings were submerged in water.
2) SL treatment (GR24): the hypocotyls of melon seedlings were submerged in 0.1 mg/L of GR24.
3) SL and IAA treatment (GR24+IAA): the hypocotyls of melon seedlings were submerged in 0.1 mg/L of GR24 and 250 mg/L of IAA.

After treatment, the cut hypocotyls of 12 plants in each treatment were placed in liquid nitrogen for RNA-Seq, and 30 plants from each treatment group were cultured in half-strength Murashige and Skoog medium for AR formation.

### Analysis of AR growth

After 6, 8, and 10 days, the number, length, surface area, and volume of ARs were measured by the MICROTEK scanner (MRS-9600TFU2L, MICROTEK, Hsinchu, Taiwan).

### RNA extraction and RNA-Seq analysis

The total RNA of melon ARs of different treatments (mixing three seedlings per replicate) was extracted. The quality of each RNA sample was evaluated on 1% agarose gels. RNA purity was determined by the NanoPhotometer^®^ spectrophotometer (Implen, CA, USA), and the RNA integrity was checked by an Agilent 2100 Bioanalyzer (Agilent Technologies, CA, USA).

The sequencing libraries were obtained by the Illumina HiSeq 4000 platform. The low-quality reads (over 50% Qphred values ≤20 bases in a read) and raw reads with adaptor and unknown nucleotides were removed; the filtered clean reads were mapped to the reference genome of *C. melon* (http://cucurbitgenomics.org/v2/organism/23).

Fragments per kilobase of transcript sequence per million base pairs sequenced (FPKM) were used to calculate the gene expression levels and the principal component analysis (PCA). DEGs were determined using the NOISeq method (R/Bioc package) with a Noisy Distribution Model and shown by a volcano diagram. The screening criteria for DEGs were *p*-value ≤0.05 and |log_2_Fold change| ≥ 1.0. Gene Ontology (GO) enrichment analysis of DEGs was carried out. GO terms with *p*-values lower than 0.05 were significantly enriched in DEGs. KOBAS software was used to determine the statistical enrichment of DEGs in the Kyoto Encyclopedia of Genes and Genomes (KEGG) enrichment analysis of DEGs.

### Gene expression analysis of RNA-Seq data

To validate the accuracy of transcriptome profiling, total RNA from different treatments was used for cDNA synthesis. A first-strand reverse transcriptase kit (Takara, Japan) was used to synthesize first-strand cDNA using total RNA as a template. qRT−PCR was performed on the CFX Connect Real-Time PCR Detection System (Bio-Rad, Hercules, CA, USA) using ChamQ Universal SYBR q-PCR Master Mix (Vazyme Biotech Co., Ltd., Nanjing, China). The specific primers for 13 genes were designed using Primer Premier 5.0 (**Table S1**), and *CmActin* (*MELO3C008032*) was used as the internal reference gene. Three biological replicates were performed. Relative expression levels were calculated using the 2^−ΔΔCT^ method.

### Auxin, GA, ZT, and ABA quantification

After 6, 8, and 10 days of treatment, 1 g of melon ARs was ground into powder using liquid nitrogen. Then, 8 ml of 80% precooled methanol was added, and the sample was extracted for 12 h at 4°C in the dark and centrifuged for 15 min. The supernatant was transferred, nitrogen was added to cool the aqueous phase base at pH = 8.0, 0.2 g of polyvinylpolypyrrolidone (PVPP) was added to remove impurities, and the sample was centrifuged for 5 min. The supernatant was added to 3 ml of ethyl acetate for extraction, dried with nitrogen, redissolved in 2 ml of 20% methanol, poured into a C18 column for purification, and passed through a 0.22-μm filter for membrane filtration. A 10-μl aliquot was subjected to analysis on an Agilent 1260 high-performance liquid chromatograph (with an ultraviolet detector), and ZORBAX Eclipse Plus C18 columns (5 μm, 250 × 4.6 mm) were used for reversed-phase high-performance liquid chromatography. The detection wavelength was 254 nm, the column temperature was 30°C, and the flow rate was 0.5 mg/L. The endogenous hormone level was calculated by the external standard method.

### Statistical analysis

The data were analyzed by SPSS 20.0 software, using the one-way method ANOVA.

Duncan’s multiple intervals were used to analyze significant differences, and differences were considered statistically significant at a level of *p*-values <0.05.

## Results

### Effects of GR24 and IAA on ARs in melon

**Figure 1:**
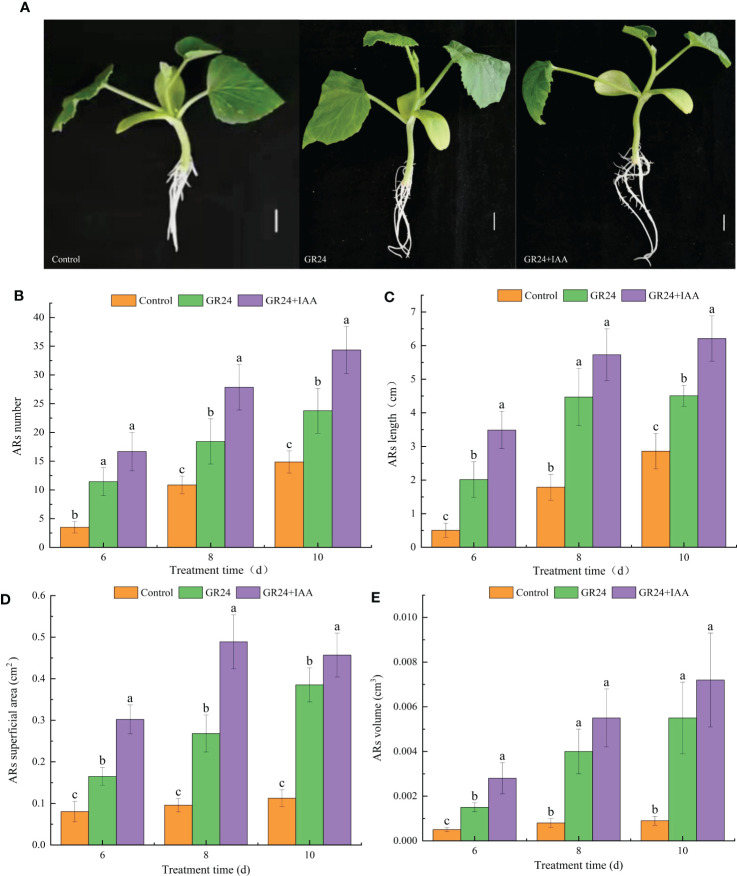
Effects of GR24 and IAA on ARs in melon. **(A)** Phenotypes of ARs under control, GR24, and GR24+IAA treatments. Bar = 1 cm. **(B)** AR number, **(C)** AR length, **(D)** AR superficial area, and **(E)** AR volume. Error bars show the standard error value. ARs, adventitious roots. The different letters represent significant differences (P < 0.05).

As shown in **Figure 1A**, the GR24 treatment and GR24+IAA treatment significantly affected AR growth. The AR number under the GR24 treatment was 1.60–3.27 times higher than that in the control group at 6–10 days. The GR24+IAA treatment further increased the AR number to 1.44–1.51 times that of the GR24 treatment group at 6–10 days (**Figure 1B**). The GR24 treatment significantly increased the AR length to 1.58–3.99 times greater than that of the control group at 6–10 days. Moreover, the AR length under the GR24+IAA treatment was 1.28–1.73 times higher than that of the control group at 6–10 days (**Figure 1C**). At 6–10 days, the AR superficial area of the GR24 treatment group was 2.06–3.42 times greater than that of the control group, and that under the GR24+IAA treatment was 1.19–1.83 times greater than that of the GR24 treatment group (**Figure 1D**). The GR24 treatment significantly increased the AR volume at 6–10 days to 3.00–6.11 times the volume of the control group. Moreover, under the GR24+IAA treatment, the AR volume was 1.31–1.87 times higher than that under the GR24 treatment at 8–10 days (**Figure 1E**). The results showed that GR24 promoted the growth of melon ARs and that GR24+IAA further promoted their growth.

### Mapping and quantitative assessment of Illumina sequencing data

A total of 449,208,638 raw reads were produced, 449,118,796 qualified reads were obtained, 419,672,281 reads (approximately 95.2%) were mapped, and 411,466,233 reads (approximately 93.4%) were uniquely mapped to the melon reference genome. Over 97.72% of the clean reads were at the Q20 level, and over 93.64% of the clean reads were at the Q30 level (**Table S2**). PCA showed the grouping of each treatment, and different treatments were separated from each other in the PCA plot (**Figure S1**). The results suggest that the transcriptome data can be used for subsequent analysis.

### Identification of DEGs responsive to the different treatments

**Figure 2:**
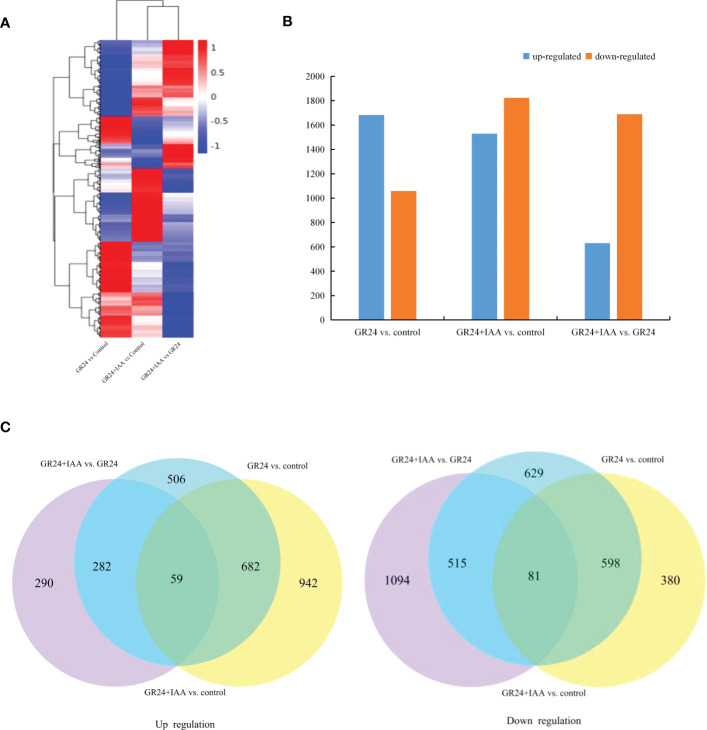
Differential gene expression in melon roots under the different treatments. **(A)** The heatmap of expression profiles of genes under the different treatments. **(B)** Numbers of upregulated and downregulated genes identified from the different comparisons. **(C)** Venn diagrams of the upregulated and downregulated genes in the three groups.

Global comparisons of the gene expression profiles of ARs under different treatments are shown in a heatmap (**Figure 2A**). The GR24 *vs.* control group comparison identified 2,742 DEGs, which included 1,683 upregulated and 1,059 downregulated genes. The GR24+IAA *vs.* control group comparison identified 3,352 DEGs, which included 1,529 upregulated and 1,823 downregulated genes, and the GR24+IAA *vs.* GR24 group comparison identified 2,321 DEGs, which included 631 upregulated and 1,690 downregulated genes (**Figure 2B**). A total of 59 upregulated genes and 81 downregulated genes were obtained in all three comparisons (GR24+IAA *vs.* control group, GR24+IAA *vs.* control group, and GR24+IAA *vs.* GR24 group) (**Figure 2C**). The above results indicated the existence of differences between the different treatments.

### GO enrichment analysis

**Figure 3:**
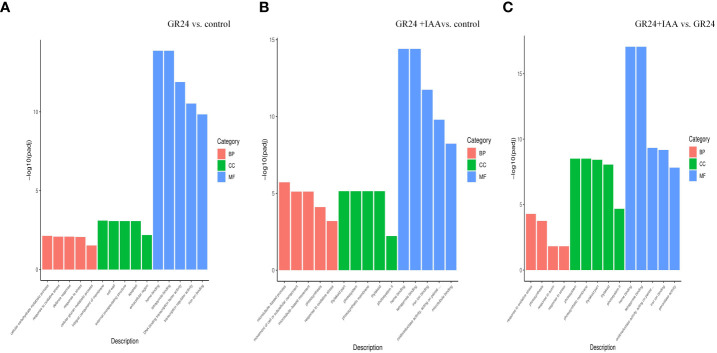
GO enrichment analysis. Classification of the GO terms enriched in the DEGs identified from the GR24 *vs.* control treatment **(A)** and GR24+IAA *vs.* control treatment **(B)**. GR24+IAA *vs.* GR24 treatment **(C)** comparisons. GO, Gene Ontology; DEGs, differentially expressed genes.

Of the DEGs identified from the GR24 *vs.* control group, 2,346 were significantly enriched in 55 GO terms, which included nine biological process (BP), five cellular component (CC), and 41 molecular function (MF) terms. Among the BP terms, “cellular carbohydrate metabolic process”, “response to oxidative stress”, and “defense response” were the top three most enriched GO terms. Among the CC terms, “integral component of membrane”, “cell wall”, “external encapsulating structure”, “apoplast”, and “extracellular region” were significantly enriched. Among the MF terms, “heme binding”, “tetrapyrrole binding”, and “DNA binding transcription factor activity” were the top three most enriched GO terms (**Figure 3**). Of the DEGs identified from the GR24+IAA *vs.* control group comparison, 2,859 were significantly enriched in 58 GO terms, which included 16 BP, eight CC, and 34 MF terms. Among the BP terms, “microtubule-based process”, “movement of cell or subcellular component”, and “microtubule-based movement” were the top three most enriched GO terms. Among the CC terms, “thylakoid part”, “photosystem”, and “photosynthetic membrane” were significantly enriched, and “heme binding” and “tetrapyrrole binding” were the most significantly enriched MF terms. Of the DEGs identified from the GR24+IAA *vs.* GR24 group comparison, 1,938 were significantly enriched in 37 GO terms, which included four BP, 14 CC, and 19 MF terms. Among the BP terms, “response to oxidative stress”, “photosynthesis”, “response to auxin”, and “response to stress” were significantly enriched, and the CC terms “thylakoid”, “thylakoid part”, “photosynthetic membrane”, and “photosystem”, as well as others, were significantly enriched. Among the MF terms, “heme binding” showed the most significant enrichment.

### KEGG enrichment analysis

**Figure 4:**
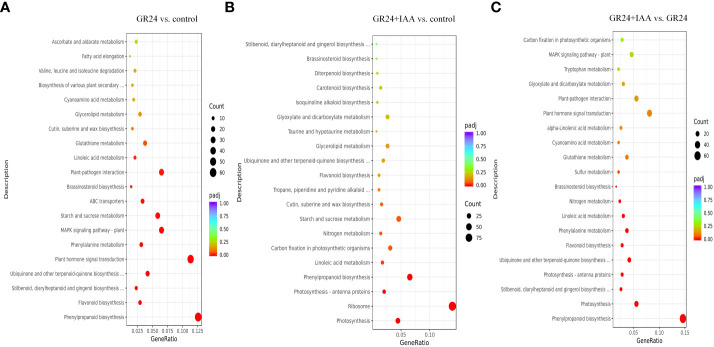
KEGG enrichment analysis. Classification of the pathways enriched in DEGs identified from the **(A)** GR24 *vs.* control, **(B)** GR24+IAA *vs.* control, and **(C)** GR24+IAA *vs.* GR24 comparisons. KEGG, Kyoto Encyclopedia of Genes and Genomes; DEGs, differentially expressed genes.

The KEGG analysis of 476 DEGs identified from the GR24 *vs.* control group comparison revealed enrichment in 105 pathways, and 315 DEGs were significantly enriched in 13 pathways, including “phenylpropanoid biosynthesis”, “plant hormone signal transduction”, and “brassinosteroid biosynthesis”. The KEGG analysis of 674 DEGs identified from the GR24+IAA *vs.* control group comparison showed enrichment in 108 pathways, and 256 DEGs were significantly enriched in eight pathways, including “photosynthesis”, “ribosome”, and “phenylpropanoid biosynthesis”. The KEGG analysis of 431 DEGs identified from the GR24+IAA *vs.* GR24 group comparison revealed enrichment in 101 pathways, and 195 DEGs were significantly enriched in 11 pathways, including “phenylpropanoid biosynthesis”, “photosynthesis”, and “brassinosteroid biosynthesis” (**Figure 4**).

### DEGs related to auxin biosynthesis and signaling pathways

**Figure 5:**
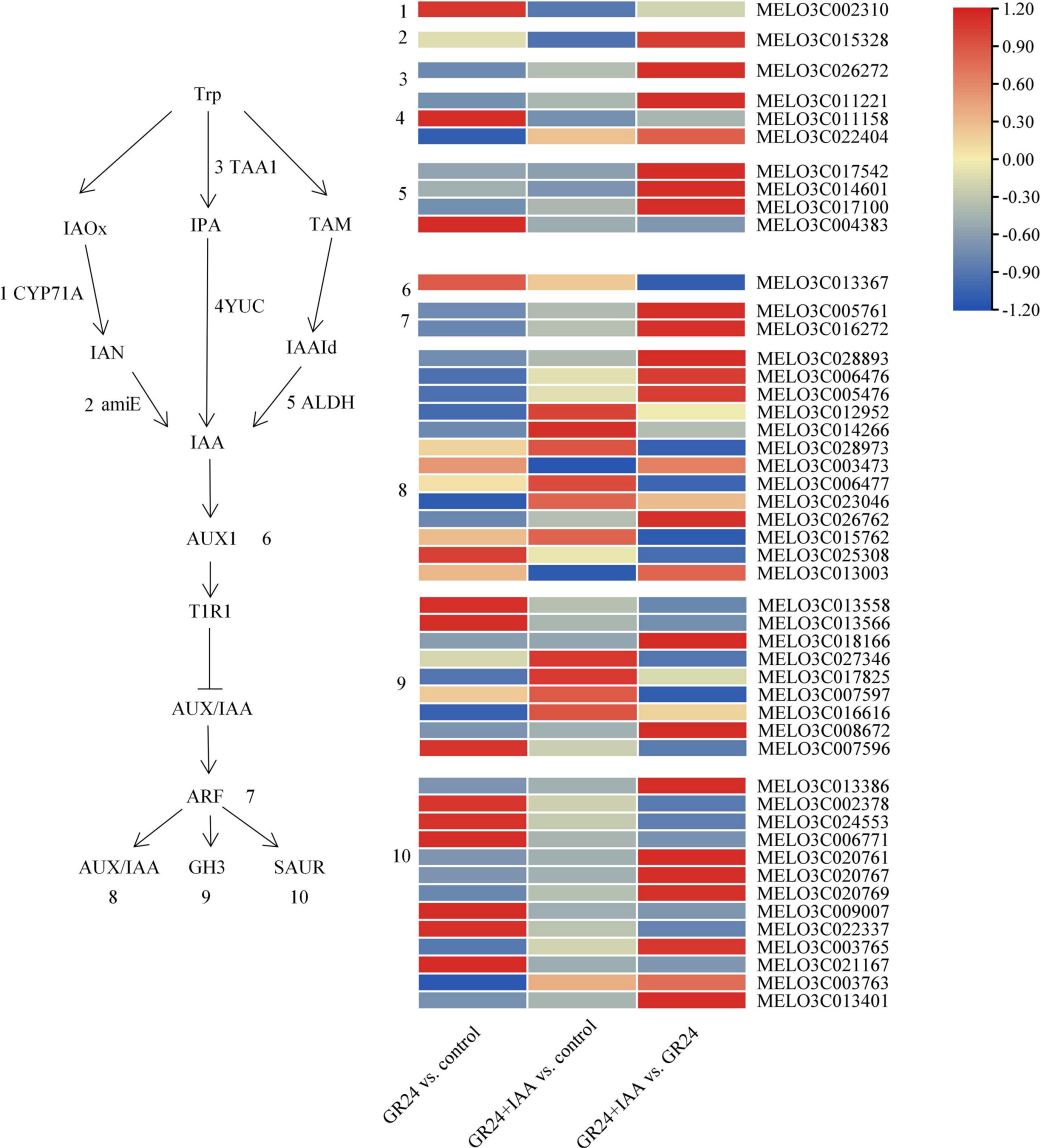
Overview and heatmap of the auxin synthesis and signaling pathway.

To explore the responses of melon roots to the GR24+IAA treatment, the genes involved in the auxin synthesis and signaling pathways were changed (**Figure 5**).

Most auxin synthesis genes were upregulated in the GR24 *vs.* control group and GR24+IAA *vs.* GR24 group. *CYP71A* was upregulated in the GR24 *vs.* control group and downregulated in the GR24+IAA *vs.* control group. *amiE* was downregulated in the GR24+IAA *vs.* control group and upregulated in the GR24+IAA *vs.* GR24 group. *TAA1* was downregulated in the GR24 *vs.* control group and the GR24+IAA *vs.* control group. Two *YUC* genes were downregulated in the GR24 *vs.* control group, one *YUC* gene was upregulated in the GR24 *vs.* control group, and two *YUC* genes were upregulated in the GR24+IAA *vs.* GR24 group. Three *NADH* genes were downregulated in the GR24 *vs.* control group, and four *NADH* genes were downregulated in the GR24+IAA *vs.* control group. In the auxin signaling pathway, some of the genes were upregulated in the GR24 *vs.* control group and the GR24+IAA *vs.* control group. *AUX1* was upregulated in the GR24 *vs.* control group and the GR24+IAA *vs.* control group and downregulated in the GR24+IAA *vs.* GR24 group. Two *ARFs* were downregulated in the GR24 *vs.* control group and the GR24+IAA *vs.* control group. The expression of 13 *AUX/IAA* genes was altered: seven *AUX/IAA* genes were downregulated in the GR24 *vs.* control group, whereas the others were upregulated, and seven *AUX/IAA* genes were upregulated in the GR24+IAA *vs.* GR24 group. The expression of nine *GH3* genes was altered: four *GH3* genes were upregulated in the GR24 *vs.* control group and the GR24+IAA *vs.* control group, and three *GH3* genes were upregulated in the GR24+IAA *vs.* GR24 group. The expression of 13 *SAUR* genes was altered under the different treatments: six *SAUR* genes were upregulated in the GR24 *vs.* control group, and seven *SAUR* genes were upregulated in the GR24+IAA *vs.* GR24 group. The results showed that auxin may be involved in the process of AR formation.

### DEGs related to SL biosynthesis and signaling pathways

**Figure 6:**
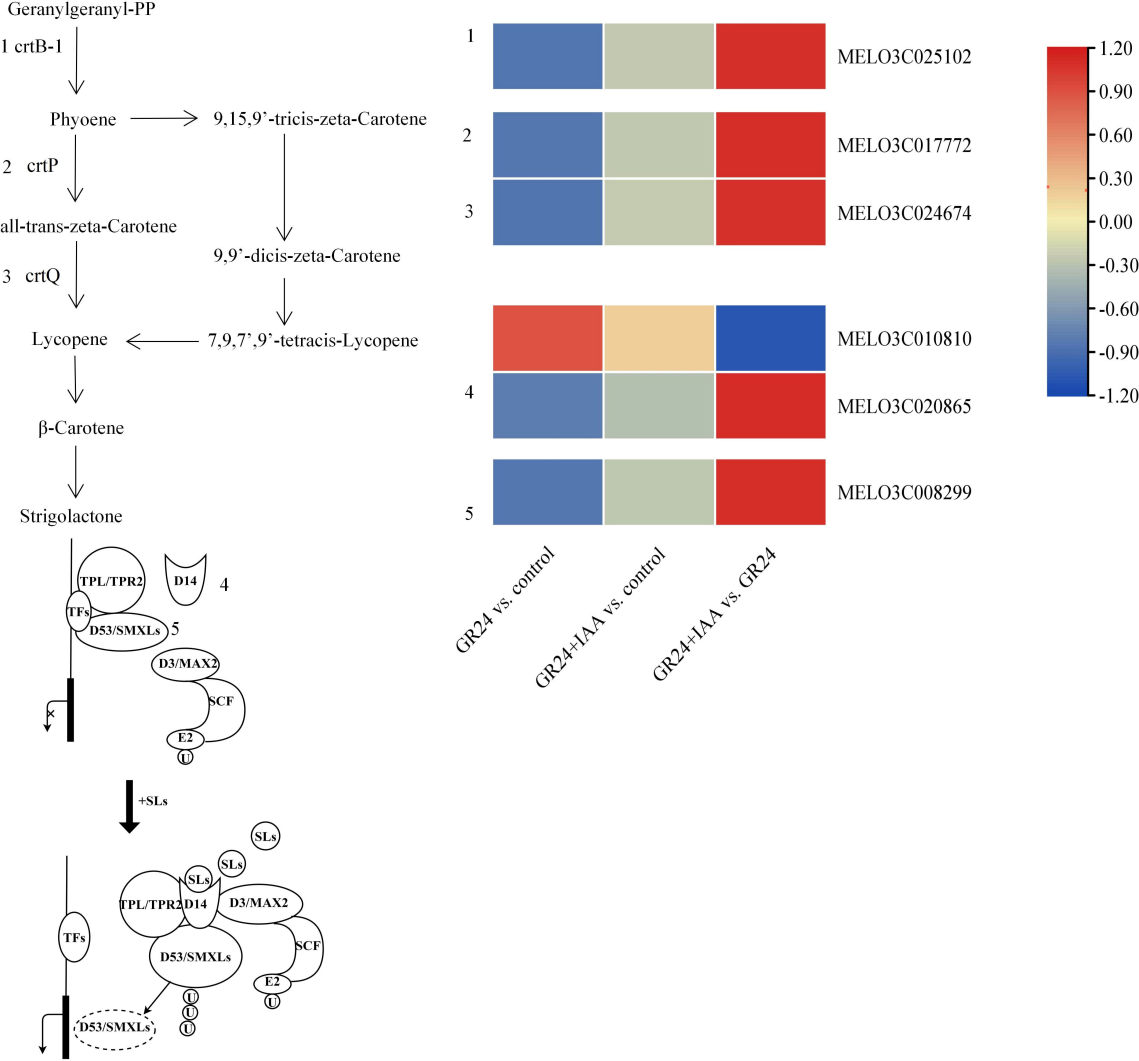
Overview and heatmap of the SL synthesis pathway and signaling pathway. SL, strigolactone.

The genes involved in SL synthesis and signaling pathways were changed (**Figure 6**). Three SL synthesis genes, *crtB-1*, *crtP*, and *crtQ*, were downregulated in the GR24 *vs.* control group and the GR24+IAA *vs.* control group and upregulated in the GR24+IAA *vs.* GR24 group. In the SL signaling pathway, the expression of two *D14* genes was changed: one *D14* gene was upregulated in the GR24 *vs.* control group and the GR24+IAA *vs.* control group and downregulated in the GR24+IAA *vs.* GR24 group. *SMAX* gene was downregulated in the GR24 *vs.* control group and the GR24+IAA *vs.* control group and upregulated in the GR24+IAA *vs.* GR24 group. The results showed that SL played an important role in the process of melon AR formation treated by GR24 and IAA.

### Analysis of DEGs related to other phytohormone signaling pathways

**Figure 7:**
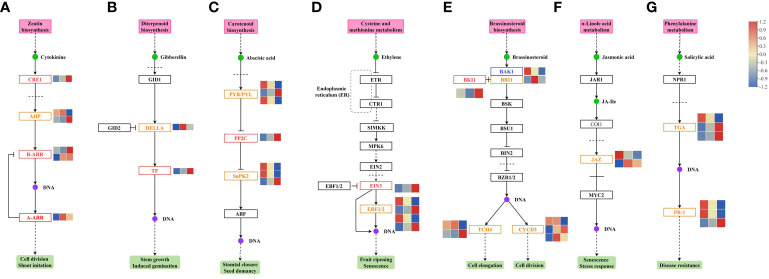
Changes the genes involved in the **(A)** CTK, **(B)** GA, **(C)** ABA, **(D)** ETH, **(E)** BR, **(F)** JA, and **(G)** SA signal transduction pathways in melon. CTK, cytokinin; GA, gibberellin; ABA, abscisic acid; ETH, ethylene; BR, brassinosteroid; JA, jasmonate; SA, salicylic acid.

RNA-Seq analysis of the different treatment groups identified the genes involved in plant hormone signal transduction pathways (**Figure 7**).

In the CTK signal transduction pathway, *CRE1*, *AHP*, *B-ARR*, and *A-ARR* gene expression was changed. *CRE1* gene was downregulated in the GR24 *vs.* control group and the GR24+IAA *vs.* control group. The expression of one *AHP* gene was upregulated in the GR24+IAA *vs.* GR24 group, and that of another gene was downregulated in the GR24+IAA *vs.* GR24 group. Two *B-ARR* genes were upregulated in the GR24+IAA *vs.* GR24 group. *A-ARR* gene was upregulated in the GR24+IAA *vs.* control group and the GR24+IAA *vs.* GR24 group (**Figure 7A**).

In the GA signal transduction pathway, two genes, *DELLA* and *TF*, showed changes in expression. The *DELLA* gene was upregulated in the GR24+IAA *vs.* control group. TF gene was downregulated in the GR24 *vs.* control group and the GR24+IAA *vs.* control group and upregulated in the GR24+IAA *vs.* GR24 group (**Figure 7B**).

In the ABA signal transduction pathway, the expression of *PYR/PYL*, *PP2C*, and *SnRK2* varied under different treatments. Two *PYR/PYL* genes were upregulated in the GR24 *vs.* control group and the GR24+IAA *vs.* control group and downregulated in the GR24+IAA *vs.* GR24 group, and another *PYR/PYL* gene was downregulated in the GR24 *vs.* control group and the GR24+IAA *vs.* control group and upregulated in the GR24+IAA *vs.* GR24 group. *PP2C* gene was downregulated in the GR24 *vs.* control group and the GR24+IAA *vs.* control group and upregulated in the GR24+IAA *vs.* GR24 group. The expression of three *SnRK2* genes was altered; two *SnRK2* genes were upregulated and one was downregulated in the GR24 *vs.* control group and the GR24+IAA *vs.* GR24 group (**Figure 7C**).

In the ETH signal transduction pathway, *EIN3* was downregulated in the GR24 *vs.* control group and the GR24+IAA *vs.* control group and upregulated in the GR24+IAA *vs.* GR24 group. Two of four *ERF1/2* genes showed downregulated expression in the GR24 *vs.* control group and the GR24+IAA *vs.* control group and upregulated expression in the GR24+IAA *vs.* GR24 group (**Figure 7D**).

In the BR signal transduction pathway, *BAK1* was upregulated in the GR24 *vs.* control group and the GR24+IAA *vs.* control group and downregulated in the GR24+IAA *vs.* GR24 group. *BKI1* was downregulated in the GR24 *vs.* control group and the GR24+IAA *vs.* control group. *BRI1* was upregulated in the GR24+IAA *vs.* control group. Two of the three *CYCD3* genes were upregulated in the GR24+IAA *vs.* GR24 group, and all three *CYCD3* genes were upregulated in the GR24+IAA *vs.* control group.

Two *TCH4* genes presented differential expression patterns (**Figure 7E**).

In the JA signal transduction pathway, only *JAZ* gene expression was altered. One *JAZ* gene was upregulated in the GR24 *vs.* control group and downregulated in the GR24+IAA *vs.* GR24 group, and another *JAZ* gene was upregulated in the GR24+IAA *vs.* control group and the GR24+IAA *vs.* GR24 group (**Figure 7F**).

In the salicylic acid (SA) signal transduction pathway, *TGA* and *PR-1* gene expression was changed. Two *TGA* genes were downregulated in the GR24 *vs.* control group and the GR24+IAA *vs.* control group and upregulated in the GR24+IAA *vs.* GR24 group. Another *TGA* gene was upregulated in the GR24 *vs.* control group and the GR24+IAA *vs.* control group and downregulated in the GR24+IAA *vs.* GR24 group. Two *TR-*1 genes were upregulated in the GR24 *vs.* control group and the GR24+IAA *vs.* control group and downregulated in the GR24+IAA *vs.* GR24 group. Another *TR-1* gene was downregulated in the GR24 *vs.* control group and the GR24+IAA *vs.* control group and upregulated in the GR24+IAA *vs.* GR24 group (**Figure 7G**).

The results showed that phytohormone-related pathways played an important role in the regulation of melon AR formation.

### Gene expression analysis of RNA-Seq data

**Figure 8:**
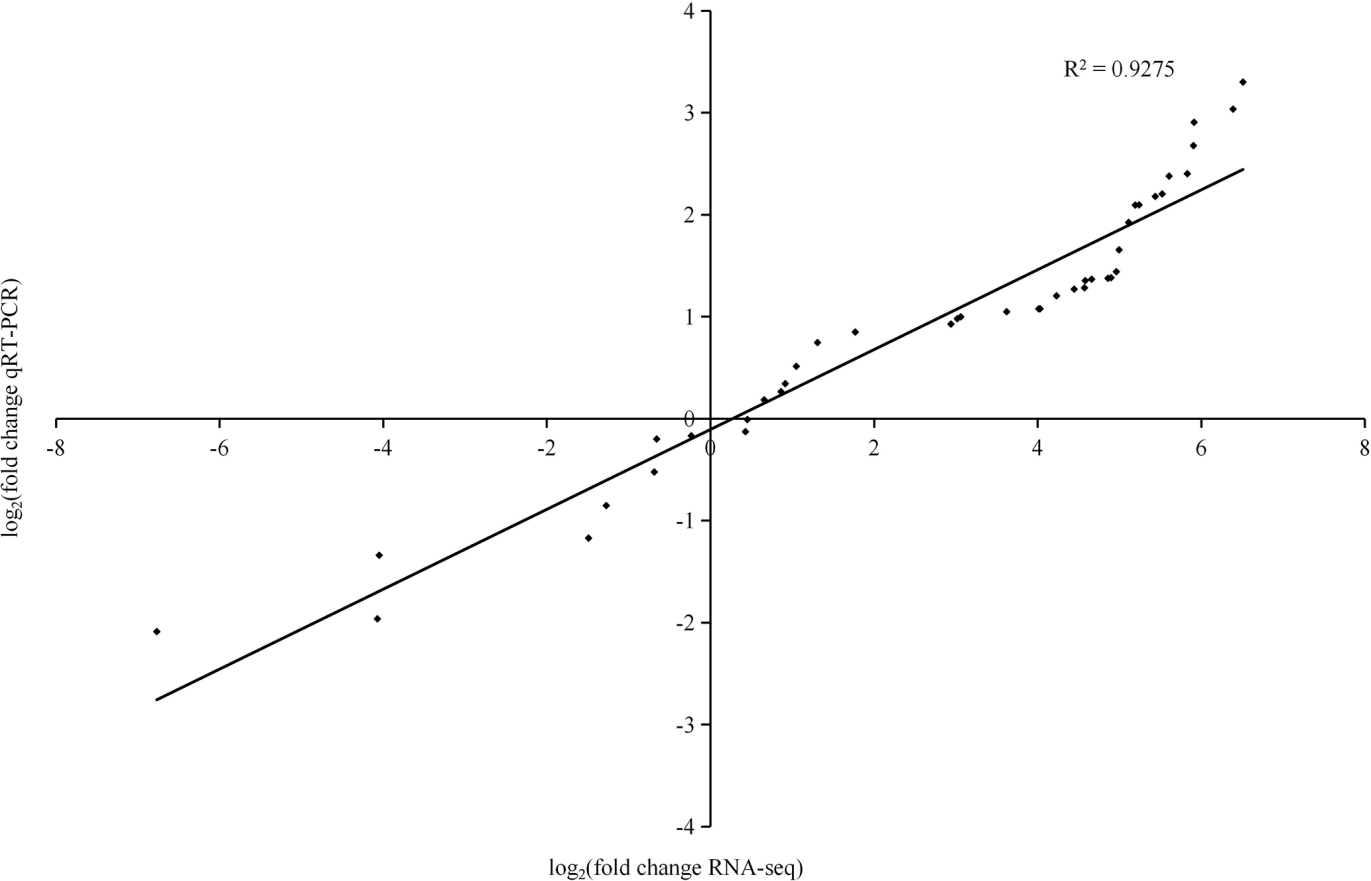
Real-time quantitative PCR validation of 13 genes. The histograms show the FPKM values determined by RNA-Seq.

To determine the reliability of our transcriptome data, 13 differentially expressed genes were randomly selected for qRT−PCR analysis (**Figure 8**). Linear regression analysis showed that the correlation coefficient was 0.9275, indicating the genes were similarly expressed by qRT−PCR and RNA-Seq.

### Changes in the phytohormone levels of ARs under different treatments

**Figure 9:**
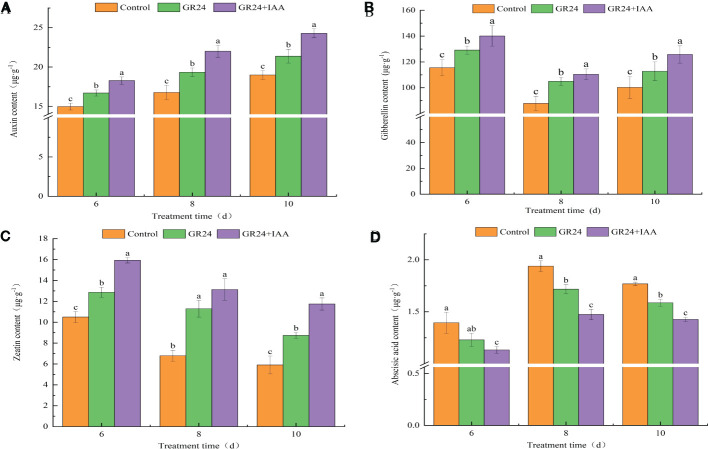
Auxin **(A)**, GA **(B)**, ZT **(C)**, and ABA **(D)** contents in ARs of melon under different treatments. GA, gibberellin; ZT, zeatin; ABA, abscisic acid; ARs, adventitious roots. The different letters represent significant differences (P < 0.05).

Auxin and GA levels were significantly different under different treatments. At 6–10 days, compared to the control, the auxin and GA levels under the GR24 treatment were increased by 11.48%–15.34% and 11.83%–19.50%, respectively; under the GR24+IAA treatment, they were increased by 22.00%–31.20% and 21.29%–25.75%, respectively (**Figures 9A, B**). From 6 to 10 days, the zeatin (ZT) content was significantly increased by 22.52%–66.17% in the GR24 treatment group compared with that in the control group and was increased by 51.76%–98.96%, under the GR24+IAA treatment (**Figure 9C**). At 6–10 days, the ABA content decreased by 10.30%–11.83% under the GR24 treatment and decreased by 18.78%–24.00% under the GR24+IAA treatment compared with the control (**Figure 9D**). The results showed that the GR24 treatment and GR24+IAA treatment increased the auxin, GA, and ZT contents and decreased the ABA content.

## Discussion

### SL regulates plant root growth

Phytohormones are important regulators of plant root development. SL is a new phytohormone that plays an important role in modulating the plant root system. Exogenous GR24 (synthetic SL) positively regulates the length of crown roots in rice (Arite et al., 2012). In grapevine, exogenous GR24 significantly increases the number and density of fine roots and the density of lateral roots (LRs) (Jiu et al., 2022). SL-mediated root growth and development are concentration dependent. A low concentration of GR24 significantly increases LR tip number and length, and a high concentration of GR24 decreases LR growth in rapeseed (Ma et al., 2020). The regulation of AR formation by SL is not clearly understood, and there are different conclusions about the SL-mediated promotion or inhibition of plant AR growth. The number of ARs is increased in SL-deficient mutants of *Arabidopsis* and pea, showing that GR24 suppresses AR formation (Rasmussen et al., 2012). In rice, the number of ARs is significantly increased by applying exogenous GR24 (Sun et al., 2015). Cherry rootstock plantlets treated with TIS108 (SL inhibitor) exhibit a significantly decreased number of ARs (Jiu et al., 2022). In our previous study, we also found that SLs positively affected AR growth in a concentration-dependent manner (Li et al., 2021). In this study, the GR24 treatment promoted AR formation, including increases in AR number, length, superficial area, and volume by 1.60–3.27, 1.58–3.99, 2.06–3.42, and 3.00–6.11, respectively, relative to the control at 6–10 days. RNA-Seq analysis of ARs treated with GR24 identified 2,742 DEGs in GR24 *vs.* control. KEGG enrichment analysis showed that the GR24 *vs.* control group DEGs were significantly enriched in phenylpropanoid biosynthesis and plant hormone signal transduction pathway. Our results showed that SL promotes the growth of ARs in melon.

### Auxin promotes SL-induced AR growth

The process of AR formation is not controlled by a single hormone but is under the control of mutual interactions among phytohormones. ACC (1-aminocyclopropane-1-carboxylic acid) and IBA (indole-3-butyric acid) increase AR density in wild-type and ethylene-insensitive mutant *Arabidopsis* (Veloccia et al., 2016). JAs negatively regulate AR formation in de-etiolated *Arabidopsis* seedlings, but MeJA (JA-donor methyl-jasmonate) combined with IBA+Kin (kinetin) enhances AR formation (Fattorini et al., 2018). Moreover, SL appears to regulate plant development *via* cross-talk with other phytohormones. SLs together with auxins regulate somatic embryogenesis and morphogenesis in tomatoes (Wu et al., 2017). In *Arabidopsis*, SL interacts with ETH to promote root hair growth and development (Kapulnik et al., 2011). Increasing evidence has shown that SL and auxin synergistically regulate the growth and development of root plants. *Arabidopsis* root hair length is significantly greater under combined GR24 and IAA treatments than under treatment with either GR24 or IAA alone (Kapulnik et al., 2011). Moreover, during the formation of ARs under exogenous phytohormone treatment, a large number of genes show expression changes. Melatonin induces significant AR formation in the hypocotyl of cucumber, and 1,296 DEGs were identified by comparing ARs treated with or without melatonin using RNA-Seq analysis (Wang et al., 2022). In this study, AR number, length, superficial area, and volume were increased more by the GR24+IAA treatment than by the GR24 treatment at 6–10 days, showing 1.44–1.51, 1.28–1.73, 1.19–1.83, and 1.31–1.87 times higher values, respectively. RNA-Seq analysis showed 3,352 and 2,321 DEGs in the GR24+IAA *vs.* control and GR24+IAA *vs.* GR24 comparisons, respectively. KEGG enrichment analysis showed that DEGs from the GR24+IAA *vs.* control group and GR24+IAA *vs.* GR24 group comparisons were all significantly enriched in “phenylpropanoid biosynthesis”. Our results further confirmed that SL and auxin synergistically promote AR formation in melon.

### Synergistic action of auxin and SL regulates AR growth *via* phytohormone pathways

It has been shown that phytohormone synthesis and transduction pathway genes are involved in plant root formation (Li et al., 2018). *CCD7* and *CCD8* in the SL biosynthesis pathway and axillary growth2 (max2) in the SL signaling pathway are involved in AR formation (Rasmussen et al., 2012; Urquhart et al., 2015). In addition to SL synthesis and transduction, auxin and other phytohormone pathways are also involved in AR formation (Çakir et al., 2012). Transcriptome analysis showed that genes involved in auxin biosynthesis and ethylene biosynthesis and signaling pathways were changed during AR formation in peach (Park et al., 2017). During the formation of ARs, endogenous hormone levels change in plants. AR numbers and IAA contents increase in *OsYUCCA1*-overexpressing rice plants, whereas AR numbers decrease in plants subjected to *OsYUCCA1* antisense treatment (Yamamoto et al., 2007). DEGs related to plant hormone pathways show significant changes in bitter tea ARs subjected to different light treatments, corresponding to increases in IAA, ZT, and GA_3_ contents and decreases in ABA contents during AR formation (Fu et al., 2023). A total of seven types of phytohormones, including ABA, auxin, BRs, cytokinins, ET, GAs, and JA, are involved in poplar AR formation (Zhang et al., 2019). In this study, the DEGs in phytohormone biosynthesis and signal transduction pathways were identified in the ARs of melon treated with GR24 and GR24+IAA. Auxin, GA, and ZT contents increased, and the ABA content decreased under the GR24 treatment and GR24+IAA treatment. Our study showed that SL and auxin synergistically regulated AR growth in melon *via* phytohormone pathways.

## Conclusions

In our study, the GR24 application promoted AR development in melon, and the GR24+IAA treatment further increased AR number, length, superficial area, and volume. The transcriptome data provided a basis for comprehensively understanding the gene expression profiles of ARs in melon after GR24 and IAA treatment. The GR24 treatment affected auxin and SL synthesis together with phytohormone signal transduction pathways. The GR24+IAA treatment changed the expression of genes involved in plant hormone signal transduction pathways, including auxin, BR, ETH, CK, GA, SA, and JA. Compared with the control levels, the auxin, GA, and ZT contents increased under the GR24 treatment and increased to greater degrees under the GR24+IAA treatment. The ABA content significantly decreased under the GR24+IAA treatment compared with the control. This work reveals an interaction between strigolactone and auxin in the induction of AR formation in melon seedlings by affecting gene expression related to plant hormone pathways and contents.

## Data availability statement

The original contributions presented in the study are publicly available. This data can be found here: NCBI GEO, accession GSE234393.

## Author contributions

JL and HG designed the experiments and drafted the initial manuscript. YW and YZ conducted the experiments. GL, XW, and BG revised the manuscript. MF and QZ analyzed the data. All authors contributed to the manuscript and approved the final manuscript.
