# Supplementary material for: Transcriptome analysis reveals that auxin promotes strigolactone-induced adventitious root growth in the hypocotyl of melon seedlings
Source: Front Plant Sci. 2023 Jun 12;14:1192340. doi: 10.3389/fpls.2023.1192340 (PMC10292653; doi:10.3389/fpls.2023.1192340)
Supplement: Supplementary file 1 [file DataSheet_1.pdf]

Supplementary Material

Table S1. Primer sequences for genes used for verification of bioinformatics gene expression results by quantitative real-time PCR

Gene ID		Sequence (5'->3')	Tm	GC%	Product Length/bp
MELO3C002287	F	TCCGAGGAATTTGGAGGTCG	59.46	55.00	185
	R	AAAGGAAGGTGGGTTGCCAT	59.81	50.00	
MELO3C019229	F	TCAGCCTCTCCCAGAAGACA	59.90	55.00	123
	R	CTCAGCTGCGGTTGAAGGTA	60.00	55.00	
MELO3C006464	F	AAGAGTTCATGGCCGGAGTT	59.31	50.00	177
	R	CCTGGTGACAATGGCTGTCT	59.96	55.00	
MELO3C009643	F	AATCGTCGTTACTGGGTTCGT	59.73	47.62	127
	R	TCCTGAAATCACAGCACGAATG	59.26	45.45	
MELO3C020859	F	CGTGGAGATGGTGGTTGGAA	59.96	55.00	131
	R	CTTTCCCCTCCGCCGATAAA	59.82	55.00	
MELO3C013770	F	CTGCTACTGCTGCACCTGAT	60.10	55.00	132
	R	AGCCTGGGTTTTGAGCAGTT	60.10	50.00	
MELO3C012908	F	AGATGTGAGGCTGCTGTTACT	59.09	47.62	154
	R	CAGAAAAAGAAGCTGGGAATGAATG	59.13	40.00	
MELO3C014491	F	CGTCTCCGAGACCACCAAAA	60.00	55.00	132
	R	CAGCCATGGACCTGGAAGAG	60.01	60.00	
MELO3C012540	F	CTTTTTGTTGAGCCTCGGCC	60.00	55.00	121
	R	TTGTTCTGGAGCTATCGGGA	58.10	50.00	
MELO3C027408	F	ACGTAGTCGATTTCGGTGCA	59.80	50.00	160
	R	TTGCAAGGCCCTTCAAATGC	60.00	50.00	
MELO3C007209	F	CGGGTACGAACTGGAAACCA	60.00	55.00	107
	R	GACTGTCCAGCTGCTCCATT	60.00	55.00	
MELO3C005549	F	TGCCCTTAAAATCGGCGTCT	60.00	50.00	120
	R	GCGGCTTCAAGATCAGCAAG	59.90	55.00	
MELO3C002094	F	AGAAGCAACTCAACTGGGTCA	59.51	47.62	109
	R	GCGACGACTTTCGCATTGAT	59.63	50.00	
Actin (*MELO3C008032*)	F	AGGTTGTTGCACCACCTGAA	60.00	50.00	143
	R	GAAGCACTTCCTGTGGACGA	60.00	55,00	

Table S2. Summary of sequence data

Sample name	Raw Reads	Clean Reads	Clean Base (Gb)	The proportion of Q20(%)	The proportion of Q30(%)
Control-1	48057278	47432202	7.11	98.05	94.36
Control-2	53111656	52313696	7.85	97.93	94.13
Control-3	49484066	48699242	7.3	97.88	93.99
GR24-1	46085858	45295538	6.79	97.96	94.22
GR24-2	47025944	46363808	6.95	97.86	94.01
GR24-3	46251398	44812992	6.72	97.77	94.03
GR24+IAA-1	49342004	47563088	7.13	97.84	94.05
GR24+IAA-2	50244706	49591576	7.44	98.01	94.31
GR24+IAA-3	59605728	58491580	8.77	97.72	93.64

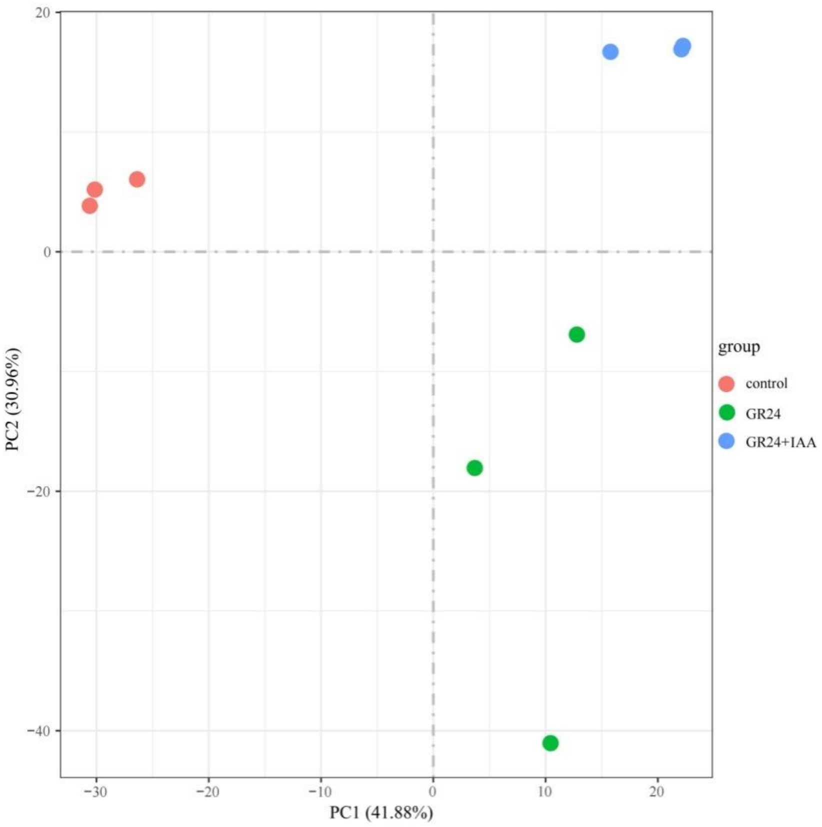

Fig S1. PCA of RNA-seq data of different treatment.
